# Current News and Opinion

**Published:** 1884-01

**Authors:** 


### PERSONAL.

Dr. George W. Field of London, has removed to No. 23 Park Street, Park Lane, W., where he will be glad to receive his American friends who are visiting England.

*Many fungi in some stage of developement *look like* bacterium termo.

### BOGUS DIPLOMAS.

Diplomas purporting to be issued by a Dental College at Wilmington, Del., have been offered for sale in London.

### Current News and Opinion.

---

### GERMAN GRADUATES.

During 1881–82, twenty-two dentists graduated in Prussia, according to official statistics.

### UNIVERSITY OF CALIFORNIA.

The second annual commencement exercises of the Dental Department of the University of California, were held with those of the Medical Department at the Baldwin Theatre, San Francisco, Cal., Tuesday evening, November 13, 1883.

The literary exercises were as follows :

Address on behalf of the Medical Department, by Prof. R. B. Kane, M. D., M. R. C. S. I.

Address on behalf of the Dental Department, by Prof. C. S. Goddard, A. M., D. D. S.

Valedictory on behalf of the Graduating Class of the Dental Department, by W. E. Price.

Conferring degrees of Doctor of Medicine, by President of University, W. I. Reid, A. M.

Conferring of degrees of Doctor of Dental Surgery, by President of University, W. I. Reid, A. M.

Administering the Hippocratic Oath to the graduates of the Medical Department, by Prof. R. Beverly Cole, A. M., M. D., M. R. C. S., England.

Address on behalf of the Alumni Association of the Dental Department, by G. W. Sichel, M. D., D. D. S.

The following candidates received the degree of Doctor of Dental Surgery :—John Nelson Blood, Charles Britton, Maria A. Burch, Edwin Overton Cochrane, Russell Hopkins Cool, Milton Francis Gabbs, William Edmund Price.

The number of matriculants for the session was twenty-six.

The next session will begin February 1, 1884, and continue till the last of October, 1884.

S. W. DENNIS, M. D.,
Dean of the Faculty.

---

### DENTAL LEGISLATION.

The first laws ever passed for the regulation of the practice of dentistry originated in France, and as a consequence, French dentists were considered superior to all others during a whole century, and all dental literature at that time was of French origin. In 1746, Fanchard published his great work on the theory and practice of Dentistry, which was the accepted standard for many years after. Many others followed during the second half of the eighteenth century. To-day, France is the only civilized country without laws relating to Dentistry, and within its boundary any individual who likes, can call himself "Dentist."—*Centralblatt fuer Zahnheilkunde.*

---

### NEW YORK ODONTOLOGICAL SOCIETY.

At the annual meeting, held during the past month, the following officers were elected for the ensuing year:

President—Wm. Jarvie, Jr.
Vice President—Wm. Carr.
Recording Secretary—E. T. Payne.
Corresponding Secretary—J. Morgan Howe.
Treasurer—Charles Miller.
Librarian—W. A. Bronson.
Curator—Jas. Goodwillie.
Executive Committe—C. A. Woodward, J. W. Clowes, W. Carr.

The meetings are held on the third Tuesday of each month.

---

### PLASTER MODELS.

Models of rare cases that it is desirable to preserve, should be made of the best plaster-of-paris, mixed with a concentrated solution of borax. They should be dried with care, and then put in a bath of parafine colored with dragons blood. The model thus prepared has the appearance of handsome Italian marble, and it may be cleaned and washed without injury. To increase the translucency more borax may be used.

To make a very hard model the plaster should be mixed with lime water, but such casts are not suitable for vulcanizing.

To increase the hardness of an already prepared cast it should be boiled in a strong solution of alum.

### POPULAR SCIENCE NEWS.

This excellent monthly, formerly *The Boston Journal of Chemistry*, has long been known as one of the very best of its class. Its every number is full of matter that is of the greatest interest to every professional man. Because of the amount of original matter that is offered to us, we are obliged to drop the Popular Science department of this Journal. To those who were interested in it, and who desire information upon the current scientific questions of the day, we cannot do a greater favor than to recommend *Popular Science News.* It is but One Dollar per year. Address it at Boston.

---

### GOOD TEETH AND GOOD INTELLECT.

The recent discussion in the French medical journals on the relation of the teeth to the brain, and their conclusions, are of importance to all brain-workers. Dr. Championniere recommends that parents and guardians should pay close attention to the teeth of those under their care, and should, when any signs of premature decay are noticeable, give their charges a holiday.—*Med. and Surg. Reporter.*

---

### DECLINED WITH THANKS.

An offer has lately been made to the municipal government at Paris, to perform the dental operations in the city hospitals free of charge. The government politely refused the offer, with the remark that "the privileges of a democratic community are to pay everybody for the work which he does."

---

### RESIGNED.

We are sorry to learn that Dr. Cravens has resigned his position as assistant upon *The Ohio Journal.* His "Varieties" gave promise of becoming a favorite department, but he retires before exhibiting to the profession all of which he was capable.

### TRACHEOTOMY FOR THE EXTRACTION OF A TOOTH FROM THE LEFT BRONCHUS.

Dr. Robert F. Weir reports in the *New York Medical Journal*, the case of a young woman who was having a tooth extracted under ether, when it slipped from the forceps and was drawn into the left bronchus. Its location could be well determined. After etherization she was turned head downwards, but this failed to dislodge the tooth. Tracheotomy was then performed, and a pair of dressing forceps, bent at four inches from the end to an obtuse angle was introduced, but the tooth could not be grasped. A long untwisted loop of slender silver wire was passed down until by good luck it came in contact with the tooth, the forceps passed over it caused it to take hold, and the tooth was removed. Rapid recovery ensued.

---

### HAY FEVER.

Dr. Carl Seiler says that all sufferers from hay fever invariably have hypertrophic nasal catarrh, and if during the winter you remove the pypertrophies with a dental drill or the cautery knife, the hay fever will not return during the summer. The anterior end of the nares is not at all sensitive, and the septum of the nose may be deflected at this point without producing any more symptoms than those caused by the occlusion of the cavity, while the posterior and middle parts are very sensitive.—*Med. and Surg. Reporter.*

---

### CARBOLIC ACID.

From the results of a series of experiments, W. Meyke arrives at the following conclusions: 1. Pure carbolic acid should be colorless, have the proper boiling point, and be entirely volatilized by heat. 2. The congealing point is of secondary importance. 3. Carbolic acid is colored red when kept in glass bottles containing lead. 4. The best vessels for keeping carbolic acid are made of tinned sheet iron.

---

### IN INDIA.

The Medical College at Madras, has added to its faculty a chair for dental surgery, with Mr. H. J. Gould, L. D. S., for Professor.

